# Insight into Highly Conserved H1 Subtype-Specific Epitopes in Influenza Virus Hemagglutinin

**DOI:** 10.1371/journal.pone.0089803

**Published:** 2014-02-26

**Authors:** Ki Joon Cho, Kwang W. Hong, Se-Ho Kim, Jong Hyeon Seok, Sella Kim, Ji-Hye Lee, Xavier Saelens, Kyung Hyun Kim

**Affiliations:** 1 Department of Biotechnology & Bioinformatics, Korea University, Sejong, Korea; 2 Antibody Engineering Laboratory, Central Research Center, Green Cross Corp., Yongin Kyunggi, Korea; 3 VIB Inflammation Research Center, Ghent, Belgium; 4 Department of Biomedical Molecular Biology, Ghent University, Ghent, Belgium; Instituto Butantan, Brazil

## Abstract

Influenza viruses continuously undergo antigenic changes with gradual accumulation of mutations in hemagglutinin (HA) that is a major determinant in subtype specificity. The identification of conserved epitopes within specific HA subtypes gives an important clue for developing new vaccines and diagnostics. We produced and characterized nine monoclonal antibodies that showed significant neutralizing activities against H1 subtype influenza viruses, and determined the complex structure of HA derived from a 2009 pandemic virus A/Korea/01/2009 (KR01) and the Fab fragment from H1-specific monoclonal antibody GC0587. The overall structure of the complex was essentially identical to the previously determined KR01 HA-Fab0757 complex structure. Both Fab0587 and Fab0757 recognize readily accessible head regions of HA, revealing broadly shared and conserved antigenic determinants among H1 subtypes. The β-strands constituted by Ser110-Glu115 and Lys169-Lys170 form H1 epitopes with distinct conformations from those of H1 and H3 HA sites. In particular, Glu112, Glu115, Lys169, and Lys171 that are highly conserved among H1 subtype HAs have close contacts with HCDR3 and LCDR3. The differences between Fab0587 and Fab0757 complexes reside mainly in HCDR3 and LCDR3, providing distinct antigenic determinants specific for 1918 pdm influenza strain. Our results demonstrate a potential key neutralizing epitope important for H1 subtype specificity in influenza virus.

## Introduction

Influenza is a viral infectious disease of the respiratory tract that affects millions of people annually [1]. Combined with subsequent infection from bacterial pneumonia, influenza remains one of the leading causes of death in many countries [2]. The 1918 influenza pandemic (pdm) killed 40–50 million people worldwide [3] and the 2009 pdm influenza that was identified in 214 countries caused more than 18,000 deaths worldwide, despite global influenza preparedness [4].

Influenza viruses which belong to the family of *Orthomyxoviridae* have three antigenically distinct types of virus, A, B, and C [5], [6]. Influenza A viruses contain three surface proteins: hemagglutinin (HA), neuraminidase (NA), and a proton channel M2 [7], [8]. The viruses are divided into subtypes on the basis of differences in the antigenicity of HA and NA. After the recent discovery of a new subtype virus genome identified from bat, there are currently 17 HA subtypes (H1–H17) and 10 NA subtypes (N1–N10) known [9], [10]. Influenza A viruses with three HA (H1, H2 and H3) and two NA (N1 and N2) serotypes have adapted to humans to produce H1N1 pdm in 1918 and 2009, H2N2 pdm in 1957, and H3N2 pdm in 1968 [11]–[13].

The 2009 pdm viruses were derived from a reassortment of six gene segments from triple reassortant swine virus and two gene segments from Eurasian influenza A (H1N1) swine virus lineage [13]. Amino acid sequence identity between 2009 pdm HA and those derived from previous vaccine strains such as A/Brisbane/59/07 (H1N1) and A/Solomon Islands/3/2006 (H1N1) reaches approximately 80%, which drops to 35–40% within the antigenic sites. It was shown that the antigenic and glycosylation patterns of 2009 pdm HA are rather similar to those of 1918 pdm HA, showing 20% amino acid difference in the antigenic sites [14].

HA is synthesized as a precursor, HA0, that trimerizes in the endoplasmic reticulum and is transferred through the Golgi apparatus to the cell surface [15]–[17]. Cleavage of the precursor HA into the subunits HA1 and HA2 by a cellular protease is required for viral infectivity [18], [19]. The HA2 stem region, proximal to the viral membrane, is highly conserved across strains and among most subtypes. Since the first cross-neutralizing antibody against influenza virus was reported [20], many structures of broadly neutralizing antibodies in complex with HA proteins have been determined [21]–[24]. Several studies also reported the existence of HA subtype-specific and inter subtype-conserved epitopes [25]–[27]. However, immune specific epitopes in H1N1 influenza virus have not been completely assessed.

During the production of H1-specific monoclonal antibodies against 2009 pdm H1N1 strains, we isolated and characterized nine H1-specific monoclonal antibodies which neutralized a broad range of H1 subtype influenza viruses. Among them, we determined the structure of the HA protein from a 2009 pandemic virus A/Korea/01/2009 (KR01) in complex with the Fab fragment from GC0587 and compared with the KR01 HA-Fab757 complex structure. In addition to GC0587, GC0757 exhibits additional activity against A/Brevig Mission/1/1918. The structural features of the complexes provide a understanding of how antibodies with subtype specificity can distinguish antigenic determinants.

## Results

### *In vivo* Production and *in vitro* Characterization of Monoclonal Antibodies

In our initial immunogenicity experiment, A/California/07/2009 (CA07) was used to immunize groups of BALB/c mice and antibody-producing cells were screened by ELISA for secretion of antibodies. Nine monoclonal antibodies were then tested for neutralization against a panel of 14 isolates including H1, 2009 pdm H1, H2, H3, H5, and H7 (Table 1). Among them, four monoclonal antibodies GC0587, GC0757, GC1517, and GC1761 were shown to neutralize a broad range of H1 subtype influenza A viruses including 2009 pdm isolates. GC0587, GC0757 and GC1517 exhibited significant neutralizing activity against 2009 pdm H1N1 strains and a seasonal H1N1 strain, whereas GC0757, GC1517 and GC1761 showed the activity against A/Brevig Mission/1/1918.

**Table 1:** In vitro neutralization activity of antibodies against a panel of HA from influenza A viruses.

Antibody HA from Strains*	GC 0346	GC 0352	GC 0587	GC 0757	GC 1245	GC 1289	GC 1517	GC 1747	GC 1761
A/Brisbane/59/2007(H1N1)	−	−	++	++	−	−	++	−	+
A/California/04/2009(H1N1)	++	+	++	++	−	−	++	−	++
A/Brevig Mission/1/1918(H1N1)	−	−	−	++	−	−	++	−	++
A/California/07/2009(H1N1)	++	++	++	++	−	−	++	−	++
A/Japan/305/1957(H2N2)	−	−	−	−	−	−	++	−	−
A/Brisbane/10/2007(H3N2)	−	−	−	−	−	−	−	−	−
A/Anhui/1/2005(H5N1)	−	−	−	−	−	−	−	−	−
A/Indonesia/5/2005(H5N1)	−	−	−	−	−	−	−	−	−
A/Viet Nam/1194/2004(H5N1)	−	−	−	−	−	−	−	−	−
A/Viet Nam/1203/2004(H5N1)	−	−	−	−	−	−	−	−	−
A/bar-headed goose/Qinghai/14/2008 (H5N1)	−	−	−	−	−	−	++	−	−
A/turkey/Turkey/1/2005(H5N1)	−	−	−	−	−	−	−	−	−
A/Netherlands/219/03(H7N7)	−	−	−	−	−	−	−	−	−
2009 pandemic vaccine	++	++	++	++	++	++	++	+	++

+represents a positive neutralization activity of each antibody determined by ELISA and –represents a negative neutralizing activity.

*A/California/04/2009(H1N1), A/Brisbane/59/2007(H1N1), A/California/04/2009(H1N1), A/Brevig Mission/1/1918(H1N1), A/California/07/2009(H1N1), A/Japan/305/1957(H2N2), A/Brisbane/10/2007(H3N2), A/Anhui/1/2005(H5N1), A/bar-headed goose/Qinghai/14/2008(H5N1), A/Indonesia/5/2005(H5N1), A/turkey/Turkey/1/2005(H5N1), A/Viet Nam/1194/2004(H5N1), A/Viet Nam/1203/2004(H5N1), A/Netherlands/219/03(H7N7).

**Figure 1:**
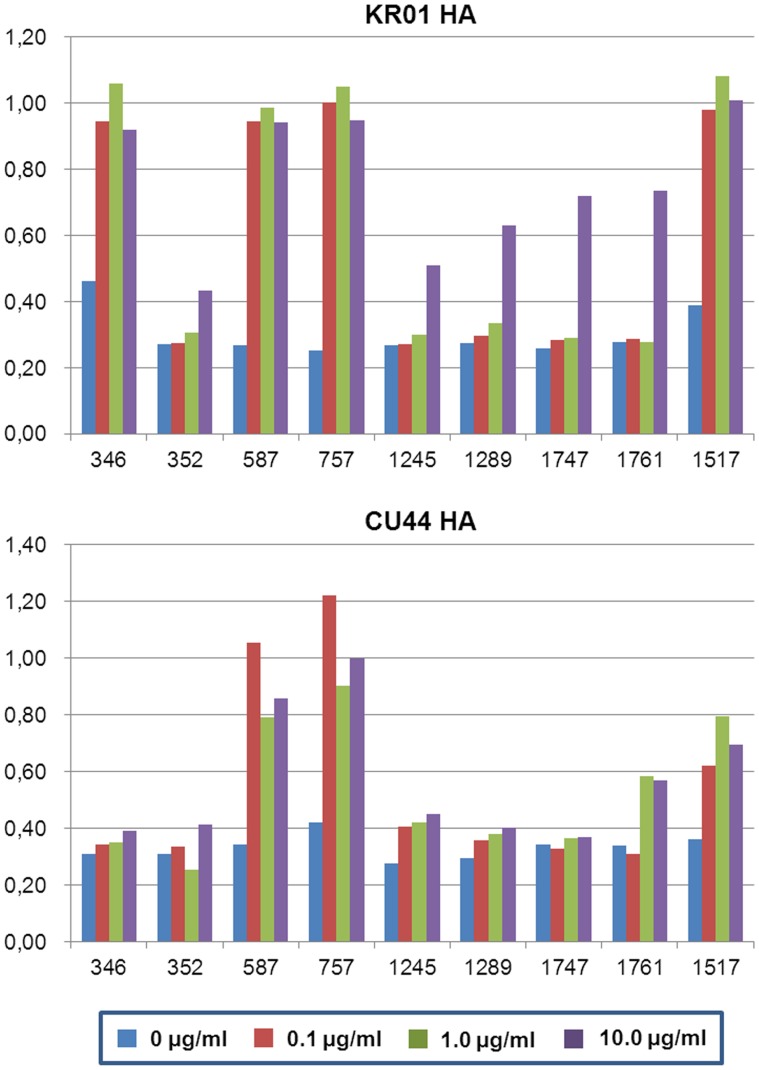
Binding affinities of monoclonal antibodies against H1 HAs. ELISA analysis results of monoclonal antibodies against KR01 and CU44 HAs that were coated on the 96-well plates to which 0 to 10 µg/ml of antibodies were added. Absorbance was measured after addition of TMB solution to each well at 490 nm.

Examination based on ELISA assays for the antigenicity against KR01 HA revealed that GC0346, GC0587, GC0757, and GC1517 had significantly high affinity to HA (Fig. 1), whereas GC0352, GC1245, GC1289, GC1747, and GC1761 showed moderate or little affinity even at high concentrations. In particular, GC0587 and GC0757 showed high affinity binding to HAs derived from both KR01 and CU44 strains and their Fab fragments were found to bind to HAs derived from H1 subtype viruses with high affinities (K_d_ 2.5–8.9 nM). The dissociation constants were within the range typically required for effective neutralization, comparable to stem binding antibodies (K_d_ ∼10 nM) [21]. In order to study broad antigenicity among H1 subtype strains, we selected four antibodies GC0346, GC0587, GC0757, and GC1517 and attempted to crystallize their Fab fragments in complex with KR01 HA. Our attempt yielded crystals derived from antibodies GC0587 or GC0757 only, suitable for X-ray structural determination.

### Overall Structure of the KR01 HA-Fab0587 Complex

**Figure 2:**
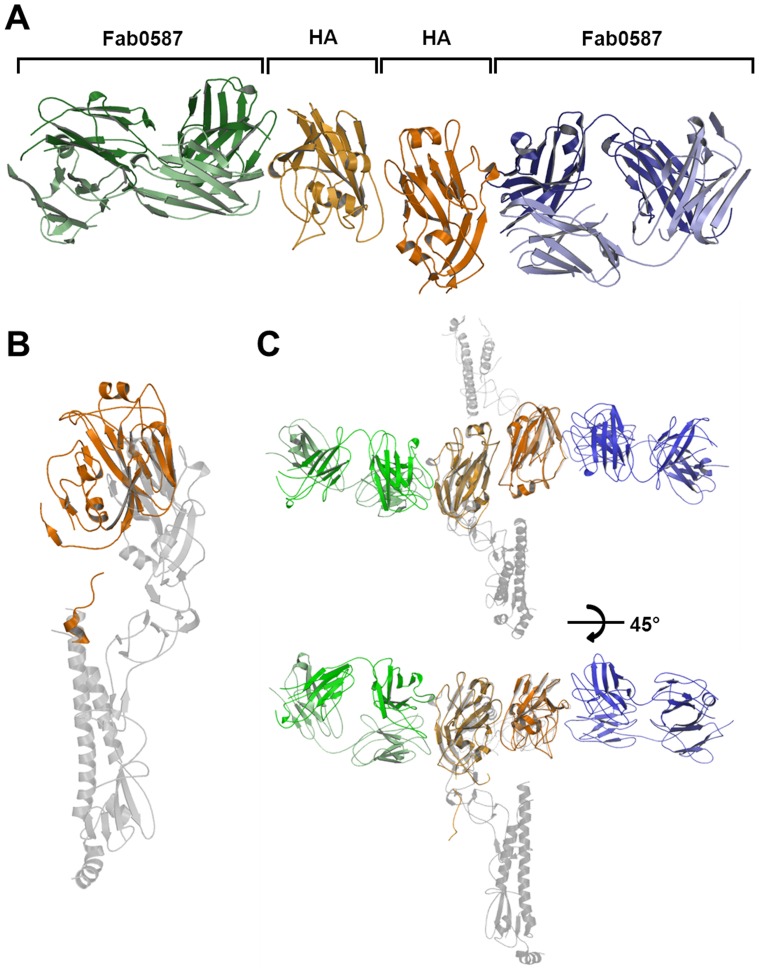
Overview of the structures of KR01 HA and its complex with Fab0587. (A) Structure of KR01 HA-Fab0587 complex. HA are colored in orange and light brown, H-chains are blue and green, and L-chains are light blue and lime. (B) Superposition of KR01 HA in Fab0587 complex and head domain of KR01 HA structures, and (C) superposition of stem region between Fab0587 bound KR01 HA and Free KR01 HA. Gray color represents free HA, and orange color represents HA in complex.

The purified recombinant KR01 HA was monomeric in solution. The structure from small rhombic crystals (0.02×0.02×0.05 mm) of KR01 HA-Fab0587 complex was determined at 3.1 Å resolution (Table 2). The asymmetric unit of the crystal contained four copies of HA-Fab0587 complexes, where Fab binds to the head region of KR01 HA resulting in a linear arrangement of the complex consisting of one Fab, two head regions of HA, and another Fab molecule (Fig. 2A). The HA-Fab0587 complex structure revealed head-to-head arrangement of HA as shown in KR01 HA (PDB ID: 4EDA) and the HA-Fab0757 complex (r.m.s.d. was 1.3 Å), to which the longitudinal axis of Fab in the complex was parallel. The overall arrangement of HA and Fab0587 was thus very similar to that of the HA-Fab0757 complex structure [28]. Superposition of the two complex structures yielded an overall r.m.s.d. of 2.1 Å, whereas the head or stem regions superimposed well individually (0.9 and 0.8 Å on average, respectively). The stem region was largely invisible in the electron density map, for which a partial model could be built, consisting of residues 414–427 (87–100 of HA2) in loop and α-helical conformations with high B factors ranging from 54–128 A^2^. A possible orientation of the HA2 domain can be derived from this partial model. When the KR01 HA structure was superimposed to that of the HA-Fab0587 complex, the head region of KR01 HA was found to be rotated clockwise by ∼20° relative to the stem region (Fig. 2B). We observed that there are empty spaces in the crystal lattice for which the HA2 domain can exist (Figure S1 in File S1). The conformation of the stem region is known to be metastable, which is temporarily stabilized by the head region of capped conformation [29], [30]. The conformational changes of HA that alleviate the constraints imposed in the pre-fusion state are known to provide the energy that is required to induce membrane fusion. The KR01 HA-Fab0587 complex structure demonstrates that the head region of HA molecule is stabilized by antibody binding and the invisible stem region adopts different orientations relative to the head region from what we have observed in typical HA proteins (Fig. 2C).

**Table 2:** Data and refinement statistics.

Data statistics		HA-Fab0587	
Wavelength (Å)		1,0000	
Number of reflections(unique reflections)		230,693 (51,527)	
Resolution range (Å)		50.0∼3.1 (3.2∼3.1)	
Completeness (%)		94.4 (90.0)	
R_merge_ (%)*		18.8 (52.0)	
I/sigma		6.4 (2.2)	
Space group		P2_1_	
Unit cell parameters		a = 27.7 Å, b = 237.9 Å, c = 94.3 Å	
		α = 90°, β = 110.3°, γ = 90°	
**Refinement statistics**			
Resolution range (Å)		40.3∼3.1	
Number of reflections		48,932	
R/R_free_ (%)**		23.8/28.3	
r.m.s. deviation			
bonds (Å)		0.013	
angles (°)		1.94	
# of water molecules		11	
Average B (Å^2^)		22.1	
**Ramachandran statistics (%)** ***			
Favoured	81.1		
Allowed	17.4		
Outlier	1.5		

*R_merge_ = Σ|I - <I>|/Σ<I>, where I and <I> are the measured and averaged intensities of multiple measurements of the same reflection, respectively. The summation is over all the observed reflections.

**R = Σ|*F*_o_ – *F*_c_|/Σ|*F*_o_| calculated for all observed data. R_free_ = Σ|*F*_o_ – *F*_c_|/Σ|*F*_o_| calculated for a specified number of randomly chosen reflections that were excluded from the refinement.

***Calculated using PROCHECK [41].

### A conserved H1 Subtype-specific Epitope of HA

**Figure 3:**
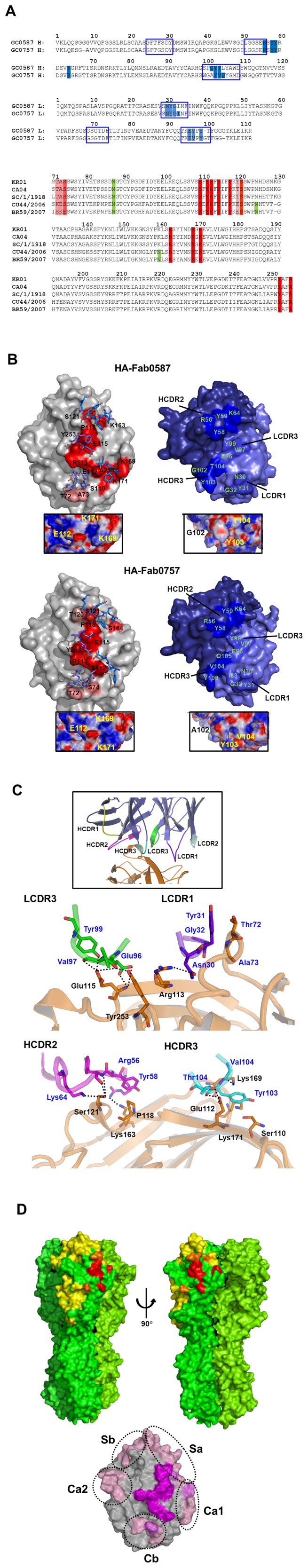
Sequence alignments and antigenic sites. (A) Sequence alignments of variable region of GC0587 and GC0757 (upper panel) and H1 HAs (lower panel). Residues in CDRs are in blue open boxes and residues that interact with HA are highlighted in blue filled boxes. Epitopes in H1 HAs are highlighted in pink, and more conserved residues are highlighted in red. Potential glycosylation sites are highlighted in green. (B) Surface representations of KR01 HAs (gray) and Fab fragments (dark blue and light blue for H-chain and L-chain, respectively). Antigenic sites are colored in red for highly conserved residues and pink for moderately conserved residues. Amino acid residues involved in the interactions between KR01 HA and Fab0587 are colored in blue and slate. Insets are surface charge representations with contours from −10 (red) to+10 (blue) kT through 0 (white). (C) Detailed interactions of HA with Fab0587. HA and Fab are colored in orange and blue, respectively. Residues that contribute to the interactions are represented as stick models. LCDR1 and LCDR3 are colored in purple and green, respectively (upper panel), and HCDR2 and HCDR3 are colored in magenta and cyan, respectively (lower panel). (D) Residues found at other structurally characterized antibody complexes are colored in yellow, those at both Fab0587 and other antibody complexes are in orange, and those against GC0587 are in red. Classical antigenic sites are colored in light pink (Ca, Cb, Sa, and Sb) and those at both Fab0587 antigenic sites are in pink.

The amino acid sequence identity between the variable domains of GC0587 and GC0757 is 93% in the H-chain and 98% in the L-chain (Fig. 3A), with the largest difference in HCDR3. The interface in the HA-Fab complexes was found to consist of interactions with heavy chain CDRs (HCDRs) 2 and 3 and light chain CDRs (LCDRs) 1 and 3 (Fig. 3B). A total buried surface area at the interface in the Fab0587 complex is 1,029 Å^2^, of which 48% arise from binding to the H-chain and 52% from the L-chain. In the Fab0757 complex, it is 993.2 Å^2^, with 50% from the H- and L-chains each. The buried surface areas are in the increasing order of LCDR1, HCDR2, LCDR3, and HCDR3, ranging from 230 to 300 Å^2^. LCDR3 and HCDR3 have the largest number of interactions in the HA-Fab0587 complex, whereas LCDR3 and HCDR2 have the largest in the HA-Fab0587 complex (Table S1 in File S1). It is notable that aromatic amino acid residues are found to contribute to the interactions at the interface between HA and Fab: Tyr31 of LCDR1, Tyr58 of HCDR2, Tyr99 of LCDR3, and Tyr103 of HCDR3 interact with residues Thr72, Lys163, Glu115, and Lys171 of HA, respectively, in both complex structures (Fig. 3C).

Human κ chain LCDR3 is typically nine amino acids long, whereas HCDR3 lengths range from 6 to 28 amino acids in adults [31]. HCDR3 and LCDR3 of 6 to 8 residues in length in Fab0587 and Fab0757 represent a short-length CDR, but they contribute substantially to the interactions with HA molecules (Fig. 3C, inset). Glu112 and Glu115 have close contacts with the main chain atoms of Tyr103 and Thr104 in HCDR3 and of Glu96 and Val97 in LCDR3, respectively. The side chain of Glu115 forms a hydrogen bond with the hydroxyl group of Tyr99. Lys169 and Lys171 also contribute significantly to the interaction in HCDR3. These interactions revealed common features at the interface in the complex structures. Both Glu112 and Glu115 are highly conserved in H1 subtype viruses, whereas they are not in other subtype HA sequences (Fig. 3A and Figure S2 in File S1). In addition, Pro118, Ser121, Lys163, and Tyr165 in HA that interact with HCDR2, and Tyr253 that interacts with LCDR3 are highly conserved in H1 subtype strains. Notably, these residues are found to be different from those of the known antigenic sites, Sa, Sb, Ca, and Cb (Fig. 3D). In this context, KR01 HA structures in complex with GC0587 or GC0757 reveal a distinct epitope in the head region which was not reported before.

### Interactions at the KR01 HA-Fab Complexes

**Figure 4:**
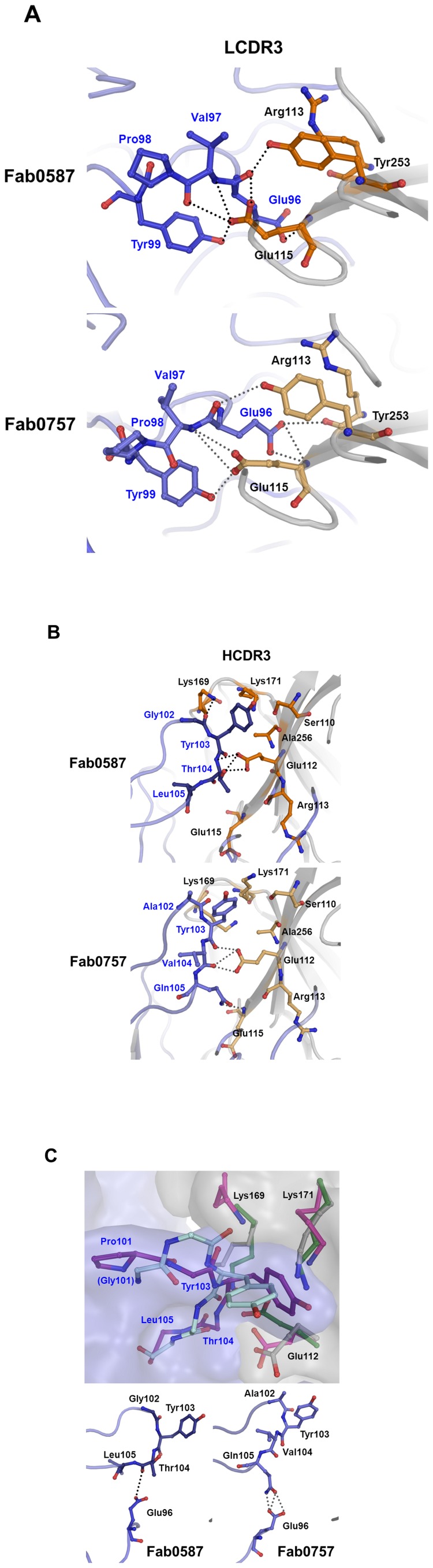
Comparison of Fab binding sites. Residues involved in interactions between HA and Fab0587 are represented as a ball-and-stick model and hydrophilic interactions as dotted line. Residues of HA in complex with Fab0587 are colored in orange and H-chains of Fab0587 and Fab0757 are in dark and light blue, respectively. (A) Comparison of LCDR3, (B) HCDR3. (C) Superposition of KR01 HA bound to Fab0587 (magenta), KR01 HA bound to Fab0757 (dark gray), free KR01 HA (yellow), 1918 pdm HA (green), Fab0587 (purple), and Fab0757 (light teal). Surface representations are based on the KR01HA-Fab0587 complex structure.

Both GC0587 and GC0757 neutralize a broad range of H1 subtype viruses including 2009 pdm H1N1 and seasonal H1N1 strains, and GC0757 further reacts with 1918 pdm H1N1 (Table 1 and Fig. 1). Despite similar amino acid sequences and common structural features of the two Fab fragments, a detailed structural analysis of the HA-Fab0587 complex revealed several epitope regions distinct from those of the HA-Fab0757 complex. First, the main chain of HA from Thr72 to Ser74 moves closer to the Fab fragment in the Fab0587 complex and interacts mostly with LCDR1 from Asn30 to Ile33 (Figure S3 in File S1). There are relatively less hydrophilic interactions between HA and LCDR1 in the Fab0587 complex than other CDRs. Second, the interactions between HA and LCDR3 are mainly mediated by Glu115 and Tyr253 of HA and Glu96 and Tyr99 of Fab (Fig. 4A). In the Fab0587 complex, the carboxylic group of Glu115 of HA interacts with the main chain atoms of Glu96 and Val97 and the hydroxyl group of Tyr99. In contrast, the main chain atoms of Arg113 and side chain atoms of Tyr253 interact with Glu96 in the Fab0757 complex. The side chain of Glu115 forms hydrogen bonds with the main chain nitrogen of Val97. In fact, the number of interactions by LCDR3 in the 757 complex is greater than that in the 587 complex (Table S1 in File S1).

Third, Arg56 of HCDR2 interacts with the side chain of Lys163 of HA in the Fab0587 complex, while it interacts with the main chain of Ser164 of HA in the Fab0757 complex (Figure S4 in File S1). Tyr58 has hydrophobic contacts with Pro118 and the backbone atom of Tyr59 has hydrophilic interactions with Ser121 of HA in both complex structures. Fourth, the main differences between Fab0587 and Fab0757 reside in HCDR3, where only three residues (Tyr103, Tyr106, and Asp109) are in common from residues 99 to 110. The side chain of Glu112 of HA interacts with Thr104 in Fab0587 (Fig. 4B). In contrast, the side chain of Glu112 of HA interacts with the main chain oxygens of Tyr103 and Val104 in Fab0757. The interaction between Glu115 of HA and Gln105 of Fab is absent in the Fab0587 complex, whereas it is present in the Fab0757 complex due to Leu to Gln mutation. Notably, the side chain of Tyr103 in the Fab0587 complex is in a favourable arrangement for a cation-π interaction with that of Lys171 of HA, and Lys169 has additional interactions with the main chain oxygen of Gly102 (Fig. 4C). However, the side chain of Tyr103 protrudes outside from the surface in the Fab0757 complex, where the cation-π interaction is absent. Both Lys169 and Lys171 are highly conserved among H1 subtype viruses and adopt distinct conformations in each complex, interacting to different HCDR3 loop conformations.

### Epitope Specificity Based on HA Structures

Both 2009 pdm KR01 HA and 1918 pdm HA exhibit 87% pairwise sequence identity, showing 20% amino acid difference in the antigenic sites. However, antigenic and glycosylation patterns of 2009 pdm HA are rather similar to those of 1918 pdm HA [14]. In the Fab0587 complex, the presence of Pro101 in HCDR3 may play an important role in stabilizing the protrusion of Tyr103 (Fig. 4C). Lys171 of HA is consequently able to make a cation-π interaction with Tyr103. In contrast, the presence of Gly101 in HCDR3 in the Fab0757 complex creates a space for the side chain of Lys169 of HA to occupy, and the cation-π interaction is absent. When the structures of KR01 HA and 1918 pdm HA are superimposed, the side chains of Lys172(Lys169) and Lys174(Lys171) of 1918 pdm HA are close to interact with Glu112. In the Fab0587 complex, however, they would not be compatible with the protruding conformation of HCDR3, whereas they can make their conformations complementary to HCDR3 in the Fab0757 complex. Our results thus suggest that the conformation of the HCDR3 backbone in the Fab0587 complex is too close to interact with 1918 pdm HA, causing steric hindrance. Tyr103 and Thr104 form hydrogen bonds with Glu96 of LCDR3. In contrast, Gln105 in Fab757 interacts with Glu96 of LCDR3 (Fig. 4C, lower panel). These interactions contribute to distinguished conformations between Fab0587 and Fab0757 complexes. Overall, our results strongly suggest that residues Ser110-Glu115, Lys169-Lys171, and Tyr253 of HA provide a H1-specific epitope and both HCDR3 and LCDR3 make major contributions to antibody-antigen interactions to achieve the epitope specificity.

## Discussion

### Implication of KR01 HA-Fab Complexes

The most effective way to protect against influenza virus infection is through vaccination. However, a good match between circulating strains and the isolates included in the vaccine is often difficult to attain due to rapid antigenic drift to evade existing antibody responses [32]. The introduction of 2009 pdm influenza virus into the human population re-emphasized the need for a systematic approach to assessing immunogenic epitopes. A typical binding of antibodies to variable positions at the epitope due to antigenic drift renders them largely strain specific and susceptible to antigenic escape.

GC0587 and GC0757 share common epitopes that are conserved in H1N1 strains, except for 1918 pdm virus. The structural features of the complexes provided a critical understanding of how antibodies with subtype specificity can distinguish antigenic determinants. It was previously reported that CH65 which binds to receptor binding pocket of HA cross-neutralizes an unusually large subset of H1N1 viruses using five of the six CDR loops [23]. Of 27 contact residues of the HA-Fab0587 complex structure, 7 core residues are >99% conserved across human H1 HA viruses: Glu112, Glu115, Pro118, Ser121, Lys163, Lys169, and Lys171. In particular, the conserved amino acid residues Glu112 and Glu115 have close contacts with HCDR3 and LCDR3, respectively. They thus comprise the epitopes recognized by both GC0587 and GC0757 and are different from the known antigenic sites, Sa, Sb, Ca, and Cb, exhibiting a distinct epitope in the head region for H1 subtype specificity. Notably, the new epitope is highly conserved among H1 influenza variants and has no glycosylation sites. It also reveals common features of interface contacts including HCDR3 and LCDR3, which explains that GC0587 and GC0757 have a broad cross-reactivity among H1 subtypes. H1 subtype-specific epitopes were recently identified by peptide scanning using libraries of overlapping peptides [33]. A new epitope (residues 58–72) was close to the link between the stem and globular head regions, which is highly conserved. Another epitope-mimic peptide (residues 158–182) that was highly immunogenic is close to what we observed in this study, where residues from Lys169 to Lys170 are included in the epitope mimic peptide. Taken together, unlike known H1 antigenic sites, the β-strands constituted by Ser110-Glu115 and Lys169-Lys170 formed H1 epitopes with distinct conformations from those of H3 HA. The backbone conformation of H1 HA was significantly different from that of H2, H5 and H3 HAs in roughly increasing order of deviation (Figure S4 in File S1). The 16 subtypes of HA segregate into two groups [7], where H1 and H3 belong to group 1 and 2, respectively. This epitope region was the most different in various subtype HAs and makes the largest difference in conformation between H1 and H3 HAs.

Moreover, the main differences between Fab0587 and Fab0757 complex structures reside mainly in HCDR3 and LCDR3, although some conformational differences are found in HCDR2 and LCDR1. It was shown that the antigenic pattern of 2009 pdm HA is similar to those of 1918 pdm HA [14]. The interaction of Lys172(Lys169) and Lys174(Lys171) with Glu112 of 1918 pdm HA is not compatible with the protruding conformation of HCDR3 of Fab0587, whereas it can be compatible with that of Fab0757. Thus, Glu112, Lys169, and Lys171 are important epitope residues to differentiate Fab0587 and Fab0757, and HCDR3 and LCDR3 make major contributions to antibody-antigen interactions to achieve epitope specificity. The unpredictability of the occurrence of influenza epidemics is originated from the wide range of antigenically different viruses, and the structural features of the complexes provide an understanding of how antibodies with subtype specificity can distinguish antigenic determinants. The results of our complex structures of a neutralizing monoclonal antibody with H1 subtype specificity can be used for potential use against influenza A viruses.

## Materials and Methods

### Preparation of Cells

MDCK cells (American Tissue Culture Type CCL-31) and Vero cells (Korean Cell Line Bank 10081) were grown in Dulbecco’s modified Eagle’s medium (DMEM, Invitrogen, USA) supplemented with 10% fetal bovine serum (FBS, Sigma, USA), 1% glutamine, and 1% penicillin-streptomycin (PS, Invitrogen, USA) under 5% CO_2_ humidified atmosphere at 37°C. The cells were passed for no more than 20 passages.

### Virus Collection and Assays

H1N1 A/Thailand/CU44/2006 (CU44), A/Brisbane/59/2007 (BR59) and 2009 pdm KR01, and H3N2 A/Gyeongnam/684/2006 (Gy684) were propagated in MDCK or Vero cells with DMEM supplemented with 0.2% BSA, 25 mM HEPES, 1% glutamine, 1% penicillin-streptomycin, and 2 µg/ml of L-(tosylamido-2-phenyl) ethyl chloromethyl ketone (TPCK)-treated trypsin (from bovine pancreas, PIERCE, USA) under 5% CO_2_ atmosphere at 37°C for 2–3 days. Virus titer was determined by using plaque assay (PFU/ml), TCID_50_, and hemagglutination assay and virus stocks were stored in deep freezer (−77°C) until experiments.

### Immunization and Production of Monoclonal Antibodies

Four-week old female Balb/c mice were immunized by intraperitoneal injection of 100 µg of 2009 pdm H1N1 A/California/04/2009 (CA04) vaccine dissolved in 100 µl PBS plus complete Freund’s adjuvant (Sigma-Aldrich, St Louis, MO, USA). Boost immunizations were given at 3 weeks post-vaccination. Animals were maintained and treated according to the Green Cross guidelines. All experiments in mice were approved by the Institutional Animal Care and Use Committee (IACUC) of the Green Cross.

Mouse spleen cells collected at 4 days after the 5th immunization boost with vaccine were fused to the sp2/0 myeloma cells. Fused cells were distributed over 96 well tissue culture plates at 2,000 cells per well in complete DMEM medium containing 100 µM hypoxanthine, 0.4 µM aminopterin, and 16 µM thymidine (HAT). With weekly replacement of medium, antibody-producing cells were screened by ELISA and subcloned by limiting dilution. Ferret anti-BR59 whole serum was used as 1/50 dilution and monoclonal antibody was used at a final concentration of 80 µg/ml. Positive clones were checked for isotype by using Iso-Gold™ rapid mouse-monoclonal isotyping test kit (Bioassay Works, Ijamsville, MD, USA) as described in the manufacturer’s protocol.

Microtiter plates were coated with 100 µl of vaccine or its equivalent HA (5 µg/ml) diluted in PBS per well at 4°C overnight, and blocked with PBS containing 1% BSA and 0.05% Tween 20 at room temperature for 1 hr. One hundred µl of hybridoma supernatant or purified antibodies per well was transferred into the ELISA plates. Binding reaction was carried out at room temperature for 2 hrs. Plates were subsequently washed four times with PBS containing 0.05% Tween 20, and 100 µl of HRP-conjugated goat anti-human Fab (Sigma-Aldrich, St Louis, MO, USA) diluted 1∶10,000 in PBS was added, and reactions were carried out at room temperature for 1 hr. Plates were washed four times, and 100 µl of tetramethyl benzidine substrate (KPL, Gaithersburg, MD, USA) per well was added. The absorbance was determined at 490 nm.

### Cloning and Baculovirus Production

HA gene were cloned downstream of the gp67 secretion signal sequence of the transfer vector pAcGP67A (BD Biosciences, MA, USA). Based on H3 numbering, the corresponding residues were 11–329 (1–327) (HA1) and 330–506 (1–176) (HA2), with a thrombin cleavage site, foldon region, and 6xHis-tag downstream of the HA gene sequence [34]. The recombinant HA proteins contained additional plasmid-encoded residues (ADPG for seasonal H1 and ADPGYLLEF for 2009 pdm H1) at their N-terminus and RSLVPR at the C-terminus. All sequences were confirmed by automated sequencing (Macrogen, Seoul, Korea). Plasmids encoding each HA gene were amplified in *E. coli* strain DH5α and then used to co-transfect Sf9 cells along with linearized baculovirus chromosomal DNA (BaculoGold; BD Biosciences, MA, USA) by the calcium transfection method. The virus was harvested after 5 days, and transfection efficiency was confirmed by PCR after extraction of DNA from 400 µl of virus (cell supernatant). This original virus stock was amplified through repeated rounds of infection.

### Protein Expression and Purification

Baculovirus containing HA gene was used to infect suspension cultures of Hi5 cells. After 3∼4 days at 28°C, the culture medium was harvested and applied to a Ni-NTA column equilibrated with working buffer (20 mM Tris-HCl, pH 8.0, 200 mM NaCl). The column was washed with buffer containing 50 mM imidazole and precursor HA protein was eluted in an imidazole gradient, which was dialysed against 10 mM Tris-HCl (pH 8.0) and 50 mM NaCl, and hydrolysed by thrombin for 12 hrs at 4°C for removal of the foldon region and 6xHis-tag. The reaction was halted by the addition of 1 mM phenylmethylsulphonyl fluoride, and the active form of HA was purified by Mono Q ion-exchange chromatography and Superdex 200HR size exclusion chromatography.

The neutralizing antibodies with activity against H1 subtype influenza viruses, GC0346, GC0587, GC0757, and GC1517, were purified using protein A columns (GE Healthcare, Piscataway, NJ, USA*).* Each was then treated with papain at a ratio of 1∶50 (w/w) for 2 hrs in PBS buffer (10 mM Na_2_HPO_4_, 1.8 mM KH_2_PO_4_, 137 mM NaCl, 2.7 mM KCl, and pH 7.4) with 5 mM EDTA and 5 mM L-cysteine, and the reaction was stopped with 30 mM crystalline iodoacetamide. The solution containing Fab fragments was dialyzed against the PBS buffer and applied to protein A column. The unbound fraction was purified by Superdex 200HR gel filtration chromatography. It was then mixed with HA at a 1∶1.5 molar ratio for 12 hr incubation, and the complex was further purified by Superdex 200HR gel filtration chromatography.

### Crystallization and Data Collection

The complex of HA and Fab0587 was screened by hanging drop vapour diffusion method. 1 µl of the 10 mg/ml complex in 20 mM Tri-HCl (pH 8.0) and 50 mM NaCl was mixed with 1 µl of screening solution of 20% PEG3350 and 200 mM potassium iodide and incubated at 4°C. After one week, small rhombic crystals were obtained. Diffraction data were collected with the crystals flash-cooled at 100 K in a stream of liquid N_2_ in the mother liquor containing 22% glycerol using synchrotron radiation sources at beamlines 5C at Pohang Light Source (Pohang, Korea). The crystals of HA-Fab0587 diffracted to 2.8 Å resolution, which belong to space group P21 with unit cell dimensions a = 72.8 Å, b = 237.8 Å, c = 94.2 Å, α = 90°, β = 110.3°, γ = 90° (Table 1). All data were processed and scaled using the HKL2000 program [35].

### Structure Solution and Refinement

The crystal structure of KR01 HA-Fab0587 was solved by molecular replacement using the KR01 HA-Fab0757 structure (PDB ID 4F15) as a template using CCP4 [36] or PHENIX [37]. The solvent content of HA-Fab0587 crystals were 65.2% using Matthews coefficient of 2.87, indicating that four complexes of HA and Fab fragments exist in the asymmetric unit. After the substitution of the Fab sequences with those of Fab0587 and manual adjustment using Coot [38], the initial solution was optimized by rigid body refinement, which produced interpretable electron density maps for the overall structure. Manual adjustment of the backbone and side chains was conducted and crystallographic refinement was carried out using the program PHENIX [37]. Difference Fourier maps, 2|Fo|-|Fc| and |Fo|-|Fc|, have been used to model the active site or loop regions. After a few rounds of model rebuilding, water molecules were added using the F_o_-F_c_ map peaks above 3.0σ, if the B factors were below 50 Å^2^ after refinement. The R_free_ value was used as an indicator to validate the water picking and refinement procedure and to guard against possible overfitting of the data [39]. R factor and R free were 0.23 and 0.27, respectively, after several round of refinement using PHENIX program. Data quality and refinement statistics for both Fab0587-HA and Fab0757-HA complexes are presented in Table 2. Stereochemical analysis of all refined structures using PROCHECK [40]. showed that there were 1.6% of outliers in the Ramachandran plot with 81.2% of favoured region among the 2,577 residues for HA-Fab0587. These outliers were located in the disordered loop regions.

### Accession Number

Data deposition−The atomic coordinates and structure factors have been deposited in the Protein Data Bank (www.rcsb.org **) with** PDB ID code 4LVH for H1N1 HA-Fab0587 structure.

## Supporting Information

File S1 **Supporting Information.** Table S1, Interactions between KR01 HA and Fab fragments. Figure S1, Crystal packing in the KR01 HA-Fab0587 complex. The head domain (HA1) of HA is shown in red color and Fab in blue in one complex, and others are in green. The stem regions (HA2) of HA could not be modeled due to weak electron density, but there are empty space for them. A partial model for HA2 is shown in the lower panel. Figure S2, Sequence alignments of HAs. Epitopes in H1 HAs are highlighted in red, and more conserved residues are highlighted in more red. Residues in other HA those are same with that of epitope in H1 HAs are indicated by red boxes. Potential glycosylation sites are highlighted in green. Figure S3, LCDR1 at Fab binding site. Residues involved in interactions between HA and Fab0587 are represented as a ball-and-stick model and hydrophilic interactions as dotted line. Residues of HA in complex with Fab0587 are colored in orange and H-chains of Fab0587 and Fab0757 are in dark and light blue, respectively. Figure S4, HCDR2 at Fab binding site. Residues involved in interactions between HA and Fab0587 are represented as a ball-and-stick model and hydrophilic interactions as dotted line. Residues of HA in complex with Fab0587 are colored in orange and H-chains of Fab0587 and Fab0757 are in dark and light blue, respectively. Figure S5, Comparison of novel H1 specific epitope regions among different subtypes. Superposition of the novel epitope conformations of H1 (blue), H2 (gray), H3 (dark gray), and H5 (green) HA. RMSD was calculated based on secondary structure matching of head domain of H1 with that of other subtypes, to show 1.19, 1.68 and 1.34 Å for H2, H3 and H5, respectively.
(DOCX)

## References

[1] Kwong JC, Stukel TA, Lim J, McGeer AJ, Upshur RE, et al (2008) The effect of universal influenza immunization on mortality and health care use. PLoS Med 5: e211. 18959473 10.1371/journal.pmed.0050211 PMC2573914

[2] Dushoff J, Plotkin JB, Viboud C, Earn DJ, Simonsen L (2006) Mortality due to influenza in the United States–an annualized regression approach using multiple-cause mortality data. Am J Epidemiol 163: 181–187. 16319291 10.1093/aje/kwj024

[3] Johnson NP, Mueller J (2002) Updating the accounts: global mortality of the 1918–1920 “Spanish” influenza pandemic. Bull Hist Med 76: 105–115. 11875246 10.1353/bhm.2002.0022

[4] WHO.

[5] Cox NJ, Subbarao K (2000) Global epidemiology of influenza: past and present. Annu Rev Med 51: 407–421. 10774473 10.1146/annurev.med.51.1.407

[6] Osterhaus AD, Rimmelzwaan GF, Martina BE, Bestebroer TM, Fouchier RA (2000) Influenza B virus in seals. Science 288: 1051–1053. 10807575 10.1126/science.288.5468.1051

[7] Gamblin SJ, Skehel JJ (2010) Influenza hemagglutinin and neuraminidase membrane glycoproteins. J Biol Chem 285: 28403–28409. 20538598 10.1074/jbc.R110.129809 PMC2937864

[8] Pielak RM, Chou JJ (2011) Influenza M2 proton channels. Biochim Biophys Acta 1808: 522–529. 20451491 10.1016/j.bbamem.2010.04.015 PMC3108042

[9] Rohm C, Zhou N, Suss J, Mackenzie J, Webster RG (1996) Characterization of a novel influenza hemagglutinin, H15: criteria for determination of influenza A subtypes. Virology 217: 508–516. 8610442 10.1006/viro.1996.0145

[10] Zhu X, Yu W, McBride R, Li Y, Chen LM, et al (2013) Hemagglutinin homologue from H17N10 bat influenza virus exhibits divergent receptor-binding and pH-dependent fusion activities. Proc Natl Acad Sci U S A 110: 1458–1463. 23297216 10.1073/pnas.1218509110 PMC3557073

[11] Taubenberger JK, Reid AH, Lourens RM, Wang R, Jin G, et al (2005) Characterization of the 1918 influenza virus polymerase genes. Nature 437: 889–893. 16208372 10.1038/nature04230

[12] Neumann G, Kawaoka Y (2006) Host range restriction and pathogenicity in the context of influenza pandemic. Emerg Infect Dis 12: 881–886. 16707041 10.3201/eid1206.051336 PMC3373033

[13] Zimmer SM, Burke DS (2009) Historical perspective–Emergence of influenza A (H1N1) viruses. N Engl J Med 361: 279–285. 19564632 10.1056/NEJMra0904322

[14] Xu R, Ekiert DC, Krause JC, Hai R, Crowe JE Jr, et al (2010) Structural basis of preexisting immunity to the 2009 H1N1 pandemic influenza virus. Science 328: 357–360. 20339031 10.1126/science.1186430 PMC2897825

[15] Gething MJ, Doms RW, York D, White J (1986) Studies on the mechanism of membrane fusion: site-specific mutagenesis of the hemagglutinin of influenza virus. J Cell Biol 102: 11–23. 3753607 10.1083/jcb.102.1.11 PMC2114034

[16] Copeland CS, Zimmer KP, Wagner KR, Healey GA, Mellman I, et al (1988) Folding, trimerization, and transport are sequential events in the biogenesis of influenza virus hemagglutinin. Cell 53: 197–209. 3359486 10.1016/0092-8674(88)90381-9

[17] Hebert DN, Foellmer B, Helenius A (1995) Glucose trimming and reglucosylation determine glycoprotein association with calnexin in the endoplasmic reticulum. Cell 81: 425–433. 7736594 10.1016/0092-8674(95)90395-x

[18] Steinhauer DA (1999) Role of hemagglutinin cleavage for the pathogenicity of influenza virus. Virology 258: 1–20. 10329563 10.1006/viro.1999.9716

[19] Klenk HD, Rott R, Orlich M, Blodorn J (1975) Activation of influenza A viruses by trypsin treatment. Virology 68: 426–439. 173078 10.1016/0042-6822(75)90284-6

[20] Okuno Y, Isegawa Y, Sasao F, Ueda S (1993) A common neutralizing epitope conserved between the hemagglutinins of influenza A virus H1 and H2 strains. J Virol 67: 2552–2558. 7682624 10.1128/jvi.67.5.2552-2558.1993 PMC237575

[21] Ekiert DC, Friesen RH, Bhabha G, Kwaks T, Jongeneelen M, et al (2011) A highly conserved neutralizing epitope on group 2 influenza A viruses. Science 333: 843–850. 21737702 10.1126/science.1204839 PMC3210727

[22] Corti D, Voss J, Gamblin SJ, Codoni G, Macagno A, et al (2011) A neutralizing antibody selected from plasma cells that binds to group 1 and group 2 influenza A hemagglutinins. Science 333: 850–856. 21798894 10.1126/science.1205669

[23] Whittle JR, Zhang R, Khurana S, King LR, Manischewitz J, et al (2011) Broadly neutralizing human antibody that recognizes the receptor-binding pocket of influenza virus hemagglutinin. Proc Natl Acad Sci U S A 108: 14216–14221. 21825125 10.1073/pnas.1111497108 PMC3161572

[24] Sui J, Hwang WC, Perez S, Wei G, Aird D, et al (2009) Structural and functional bases for broad-spectrum neutralization of avian and human influenza A viruses. Nat Struct Mol Biol 16: 265–273. 19234466 10.1038/nsmb.1566 PMC2692245

[25] Mueller M, Renzullo S, Brooks R, Ruggli N, Hofmann MA (2010) Antigenic characterization of recombinant hemagglutinin proteins derived from different avian influenza virus subtypes. PLoS One 5: e9097. 20140098 10.1371/journal.pone.0009097 PMC2816723

[26] Kaverin NV, Rudneva IA, Govorkova EA, Timofeeva TA, Shilov AA, et al (2007) Epitope mapping of the hemagglutinin molecule of a highly pathogenic H5N1 influenza virus by using monoclonal antibodies. J Virol 81: 12911–12917. 17881439 10.1128/JVI.01522-07 PMC2169086

[27] Vareckova E, Cox N, Klimov A (2002) Evaluation of the subtype specificity of monoclonal antibodies raised against H1 and H3 subtypes of human influenza A virus hemagglutinins. J Clin Microbiol 40: 2220–2223. 12037091 10.1128/JCM.40.6.2220-2223.2002 PMC130739

[28] Cho KJ, Lee JH, Hong KW, Kim SH, Park Y, et al (2013) Insight into structural diversity of influenza virus hemagglutinin. J Gen Virol. 94: 1712–1722. 10.1099/vir.0.051136-0 23636824

[29] Skehel JJ, Wiley DC (2000) Receptor binding and membrane fusion in virus entry: the influenza hemagglutinin. Annu Rev Biochem 69: 531–569. 10966468 10.1146/annurev.biochem.69.1.531

[30] Harrison SC (2008) Viral membrane fusion. Nat Struct Mol Biol 15: 690–698. 18596815 10.1038/nsmb.1456 PMC2517140

[31] Volpe JM, Kepler TB (2008) Large-scale analysis of human heavy chain V(D)J recombination patterns. Immunome Res 4: 3. 18304322 10.1186/1745-7580-4-3 PMC2275228

[32] Salzberg S (2008) The contents of the syringe. Nature 454: 160–161. 18615063 10.1038/454160a PMC2674578

[33] Zhao R, Cui S, Guo L, Wu C, Gonzalez R, et al (2011) Identification of a highly conserved H1 subtype-specific epitope with diagnostic potential in the hemagglutinin protein of influenza A virus. PLoS One 6: e23374. 21886787 10.1371/journal.pone.0023374 PMC3158760

[34] Bhardwaj A, Walker-Kopp N, Wilkens S, Cingolani G (2008) Foldon-guided self-assembly of ultra-stable protein fibers. Protein Sci 17: 1475–1485. 18535304 10.1110/ps.036111.108 PMC2525528

[35] Otwinowski Z, Minor W (1997) Processing of X-ray diffraction data collected in oscillation mode. Macromolecular Crystallography, Pt A 276: 307–326. 10.1016/S0076-6879(97)76066-X 27754618

[36] The CCP4 suite: programs for protein crystallography. Acta Crystallogr D Biol Crystallogr 50: 760–763. 15299374 10.1107/S0907444994003112

[37] Adams PD, Afonine PV, Bunkoczi G, Chen VB, Davis IW, et al (2010) PHENIX: a comprehensive Python-based system for macromolecular structure solution. Acta Crystallogr D Biol Crystallogr 66: 213–221. 20124702 10.1107/S0907444909052925 PMC2815670

[38] Emsley P, Cowtan K (2004) Coot: model-building tools for molecular graphics. Acta Crystallogr D Biol Crystallogr 60: 2126–2132. 15572765 10.1107/S0907444904019158

[39] Brunger AT (1992) Free R value: a novel statistical quantity for assessing the accuracy of crystal structures. Nature 355: 472–475. 18481394 10.1038/355472a0

[40] Laskowski RA, Moss DS, Thornton JM (1993) Main-chain bond lengths and bond angles in protein structures. J Mol Biol 231: 1049–1067. 8515464 10.1006/jmbi.1993.1351

[41] Laskowski RA, MacArthus MW, Moss DS, Thornton JM (1993) PROCHECK: a program to check the stereochemical quality of protein structures. J Appl Cryst 26: 283–291.

