# Supplementary material for: Insight into Highly Conserved H1 Subtype-Specific Epitopes in Influenza Virus Hemagglutinin
Source: PLoS One. 2014 Feb 26;9(2):e89803. doi: 10.1371/journal.pone.0089803 (PMC3935945; doi:10.1371/journal.pone.0089803)
Supplement: File S1 — Supporting Information. Table S1, Interactions between KR01 HA and Fab fragments. Figure S1, Crystal packing in the KR01 HA-Fab0587 complex. The head domain (HA1) of HA is shown in red color and Fab in blue in one complex, and others are in green. The stem regions (HA2) of HA could not be modeled due to weak electron density, but there are empty space for them. A partial model for HA2 is shown in the lower panel. Figure S2, Sequence alignments of HAs. Epitopes in H1 HAs are highlighted in red, and more conserved residues are highlighted in more red. Residues in other HA those are same with that of epitope in H1 HAs are indicated by red boxes. Potential glycosylation sites are highlighted in green. Figure S3, LCDR1 at Fab binding site. Residues involved in interactions between HA and Fab0587 are represented as a ball-and-stick model and hydrophilic interactions as dotted line. Residues of HA in complex with Fab0587 are colored in orange and H-chains of Fab0587 and Fab0757 are in dark and light blue, respectively. Figure S4, HCDR2 at Fab binding site. Residues involved in interactions between HA and Fab0587 are represented as a ball-and-stick model and hydrophilic interactions as dotted line. Residues of HA in complex with Fab0587 are colored in orange and H-chains of Fab0587 and Fab0757 are in dark and light blue, respectively. Figure S5, Comparison of novel H1 specific epitope regions among different subtypes. Superposition of the novel epitope conformations of H1 (blue), H2 (gray), H3 (dark gray), and H5 (green) HA. RMSD was calculated based on secondary structure matching of head domain of H1 with that of other subtypes, to show 1.19, 1.68 and 1.34 Å for H2, H3 and H5, respectively. (DOCX) [file pone.0089803.s001.docx]

Jan 22, 2014 (revised)

Insight into highly conserved H1 subtype-specific epitopes in influenza virus hemagglutinin

**Ki Joon Cho^1^**^*^**, Kwang W. Hong^2^, Se-Ho Kim^2**^, Jong Hyeon Seok^1^, Sella Kim^1^, Ji-Hye Lee^1^, Xavier Saelens^3,4^, and Kyung Hyun Kim^1^**^*^

^1^Department of Biotechnology & Bioinformatics, Korea University, Sejong 339-700, Korea, ^2^Antibody Engineering Laboratory, Central Research Center, Green Cross Corp. Yongin Kyunggi 446-799, Korea, ^3^VIB Inflammation Research Center, 9052 Ghent, Belgium, ^4^Department of Biomedical Molecular Biology, Ghent University, 9052 Ghent, Belgium

**Supplemental materials**

Contents

Supplementary Table S1

Supplementary Fig. S1-S5

**Table S1. Interactions between KR01 HA and Fab fragments**

1. KR01 HA-Fab0587 complex

| HA | | | L-chain | | | distance | | HA | | | L-chain | | | distance | |
| --- | --- | --- | --- | --- | --- | --- | --- | --- | --- | --- | --- | --- | --- | --- | --- |
| 113 | ARG | NH1 | 30 | ASN | N | 3.5 | LCDR1 | 115 | GLU | CD | 96 | GLU | CA | 3.55 | LCDR3 |
| 113 | ARG | NH1 | 30 | ASN | OD1 | 3.62 | LCDR1 | 115 | GLU | CD | 96 | GLU | CB | 3.31 | LCDR3 |
| 72 | THR | CA | 31 | TYR | CB | 3.43 | LCDR1 | 115 | GLU | CB | 96 | GLU | CB | 2.99 | LCDR3 |
| 72 | THR | C | 31 | TYR | CB | 3.84 | LCDR1 | 115 | GLU | CG | 96 | GLU | CB | 3.69 | LCDR3 |
| 72 | THR | CA | 31 | TYR | CG | 3.27 | LCDR1 | 115 | GLU | CB | 96 | GLU | CG | 3.69 | LCDR3 |
| 72 | THR | CA | 31 | TYR | CD1 | 3.37 | LCDR1 | 115 | GLU | CB | 96 | GLU | CD | 3.57 | LCDR3 |
| 72 | THR | CB | 31 | TYR | CD1 | 3.76 | LCDR1 | 115 | GLU | CD | 96 | GLU | C | 3.08 | LCDR3 |
| 72 | THR | CB | 31 | TYR | CE1 | 3.96 | LCDR1 | 115 | GLU | N | 96 | GLU | OE1 | 3.5 | LCDR3 |
| 72 | THR | CG2 | 31 | TYR | CE1 | 3.95 | LCDR1 | 115 | GLU | N | 96 | GLU | OE2 | 3.08 | LCDR3 |
| 72 | THR | CG2 | 31 | TYR | CZ | 3.98 | LCDR1 | 115 | GLU | OE1 | 96 | GLU | O | 3.36 | LCDR3 |
| 72 | THR | CA | 31 | TYR | CD2 | 3.88 | LCDR1 | 115 | GLU | OE2 | 96 | GLU | O | 2.79 | LCDR3 |
| 73 | ALA | N | 32 | GLY | N | 3.07 | LCDR1 | 253 | TYR | OH | 96 | GLU | O | 2.4 | LCDR3 |
| 73 | ALA | CA | 32 | GLY | CA | 3.5 | LCDR1 | 115 | GLU | OE1 | 97 | VAL | N | 2.95 | LCDR3 |
|  |  |  |  |  |  |  |  | 115 | GLU | OE2 | 97 | VAL | N | 3.35 | LCDR3 |
|  |  |  |  |  |  |  |  | 115 | GLU | OE1 | 97 | VAL | O | 3.23 | LCDR3 |
|  |  |  |  |  |  |  |  | 115 | GLU | OE1 | 99 | TYR | OH | 3.83 | LCDR3 |

| HA | | | H-chain | | | distance | | HA | | | H-chain | | | distance | |
| --- | --- | --- | --- | --- | --- | --- | --- | --- | --- | --- | --- | --- | --- | --- | --- |
| 163 | LYS | CE | 56 | ARG | CG | 3.65 | HCDR2 | 169 | LYS | NZ | 102 | GLY | O | 2.26 | HCDR3 |
| 163 | LYS | CD | 56 | ARG | CD | 3.74 | HCDR2 | 256 | ALA | CB | 103 | TYR | CB | 3.77 | HCDR3 |
| 163 | LYS | CE | 56 | ARG | CD | 2.99 | HCDR2 | 112 | GLU | CG | 103 | TYR | CD1 | 3.17 | HCDR3 |
| 163 | LYS | NZ | 56 | ARG | NH1 | 3.65 | HCDR2 | 112 | GLU | CB | 103 | TYR | CD1 | 3.96 | HCDR3 |
| 118 | PRO | CG | 58 | TYR | CD1 | 3.54 | HCDR2 | 112 | GLU | CD | 103 | TYR | CD1 | 3.31 | HCDR3 |
| 118 | PRO | CD | 58 | TYR | CD1 | 3.96 | HCDR2 | 112 | GLU | CG | 103 | TYR | CE1 | 3.65 | HCDR3 |
| 118 | PRO | CG | 58 | TYR | CE1 | 3.66 | HCDR2 | 112 | GLU | CD | 103 | TYR | CE1 | 3.98 | HCDR3 |
| 118 | PRO | CD | 58 | TYR | CE1 | 3.53 | HCDR2 | 112 | GLU | OE1 | 103 | TYR | O | 3.4 | HCDR3 |
| 118 | PRO | CD | 58 | TYR | CZ | 3.74 | HCDR2 | 110 | SER | CB | 103 | TYR | CE1 | 3.69 | HCDR3 |
| 121 | SER | OG | 59 | TYR | N | 3.71 | HCDR2 | 110 | SER | CB | 103 | TYR | CZ | 3.46 | HCDR3 |
| 121 | SER | OG | 59 | TYR | O | 3.81 | HCDR2 | 110 | SER | OG | 103 | TYR | OH | 3.54 | HCDR3 |
| 121 | SER | OG | 64 | LYS | NZ | 3.97 | HCDR2 | 171 | LYS | CE | 103 | TYR | CZ | 3.82 | HCDR3 |
|  |  |  |  |  |  |  |  | 171 | LYS | CD | 103 | TYR | CE2 | 3.61 | HCDR3 |
|  |  |  |  |  |  |  |  | 171 | LYS | CE | 103 | TYR | CE2 | 3.39 | HCDR3 |
|  |  |  |  |  |  |  |  | 171 | LYS | CD | 103 | TYR | CD2 | 3.54 | HCDR3 |
|  |  |  |  |  |  |  |  | 171 | LYS | CE | 103 | TYR | CD2 | 3.51 | HCDR3 |
|  |  |  |  |  |  |  |  | 112 | GLU | OE1 | 104 | THR | N | 2.7 | HCDR3 |
|  |  |  |  |  |  |  |  | 112 | GLU | OE2 | 104 | THR | OG1 | 2.46 | HCDR3 |
|  |  |  |  |  |  |  |  | 112 | GLU | OE1 | 104 | THR | OG1 | 2.62 | HCDR3 |

1. KR01 HA-Fab0757 complex

| HA | | | L-chain | | | distance | | HA | | | L-chain | | | distance | |
| --- | --- | --- | --- | --- | --- | --- | --- | --- | --- | --- | --- | --- | --- | --- | --- |
| 113 | ARG | NH1 | 30 | ASN | OD1 | 3.15 | LCDR1 | 113 | ARG | O | 96 | GLU | OE1 | 3.44 | LCDR3 |
| 72 | THR | CA | 31 | TYR | CB | 3.52 | LCDR1 | 115 | GLU | CB | 96 | GLU | CB | 3.41 | LCDR3 |
| 72 | THR | CA | 31 | TYR | CG | 3.61 | LCDR1 | 115 | GLU | CG | 96 | GLU | CB | 3.75 | LCDR3 |
| 72 | THR | CB | 31 | TYR | CG | 4 | LCDR1 | 115 | GLU | CD | 96 | GLU | CB | 3.23 | LCDR3 |
| 72 | THR | CA | 31 | TYR | CD1 | 3.97 | LCDR1 | 115 | GLU | CB | 96 | GLU | CG | 3.67 | LCDR3 |
| 72 | THR | CB | 31 | TYR | CD1 | 3.97 | LCDR1 | 115 | GLU | CB | 96 | GLU | CD | 3.42 | LCDR3 |
| 72 | THR | OG1 | 31 | TYR | OH | 4 | LCDR1 | 115 | GLU | N | 96 | GLU | OE1 | 3.74 | LCDR3 |
| 74 | SER | N | 31 | TYR | O | 3.27 | LCDR1 | 115 | GLU | N | 96 | GLU | OE2 | 2.86 | LCDR3 |
| 74 | SER | CB | 32 | GLY | CA | 3.57 | LCDR1 | 115 | GLU | CD | 96 | GLU | C | 3.95 | LCDR3 |
| 112 | GLU | CG | 33 | ILE | CD1 | 3.47 | LCDR1 | 115 | GLU | OE2 | 96 | GLU | O | 3.81 | LCDR3 |
| 112 | GLU | CD | 33 | ILE | CD1 | 3.47 | LCDR1 | 253 | TYR | OH | 96 | GLU | O | 3.4 | LCDR3 |
|  |  |  |  |  |  |  |  | 115 | GLU | OE1 | 97 | VAL | N | 4 | LCDR3 |
|  |  |  |  |  |  |  |  | 115 | GLU | OE2 | 97 | VAL | N | 3.87 | LCDR3 |
|  |  |  |  |  |  |  |  | 115 | GLU | OE2 | 97 | VAL | O | 3.91 | LCDR3 |
|  |  |  |  |  |  |  |  | 115 | GLU | CD | 99 | TYR | CE1 | 3.98 | LCDR3 |
|  |  |  |  |  |  |  |  | 115 | GLU | OE1 | 99 | TYR | OH | 2.31 | LCDR3 |
|  |  |  |  |  |  |  |  | 115 | GLU | OE2 | 99 | TYR | OH | 3.79 | LCDR3 |

| HA | | | H-chain | | | distance | | HA | | | H-chain | | | distance | |
| --- | --- | --- | --- | --- | --- | --- | --- | --- | --- | --- | --- | --- | --- | --- | --- |
| 164 | SER | O | 56 | ARG | NH1 | 3.14 | HCDR2 | 112 | GLU | OE1 | 103 | TYR | O | 2.93 | HCDR3 |
| 164 | SER | N | 56 | ARG | NH1 | 3.96 | HCDR2 | 112 | GLU | OE2 | 103 | TYR | O | 3.94 | HCDR3 |
| 164 | SER | N | 56 | ARG | NH2 | 3.65 | HCDR2 | 112 | GLU | OE1 | 104 | VAL | N | 3.95 | HCDR3 |
| 121 | SER | CB | 58 | TYR | CA | 3.28 | HCDR2 | 112 | GLU | OE1 | 104 | VAL | O | 3.19 | HCDR3 |
| 121 | SER | CB | 58 | TYR | CB | 3.38 | HCDR2 | 112 | GLU | OE2 | 104 | VAL | O | 2.72 | HCDR3 |
| 121 | SER | CB | 58 | TYR | CD1 | 3.88 | HCDR2 | 113 | ARG | O | 105 | GLN | OE1 | 3.96 | HCDR3 |
| 121 | SER | CB | 58 | TYR | C | 3.89 | HCDR2 | 115 | GLU | N | 105 | GLN | NE2 | 3.24 | HCDR3 |
| 118 | PRO | CG | 58 | TYR | CD1 | 3.84 | HCDR2 |  |  |  |  |  |  |  |  |
| 118 | PRO | CG | 58 | TYR | CE1 | 3.68 | HCDR2 |  |  |  |  |  |  |  |  |
| 118 | PRO | CG | 58 | TYR | CZ | 3.77 | HCDR2 |  |  |  |  |  |  |  |  |
| 121 | SER | OG | 59 | TYR | N | 2.9 | HCDR2 |  |  |  |  |  |  |  |  |
| 121 | SER | OG | 59 | TYR | O | 2.78 | HCDR2 |  |  |  |  |  |  |  |  |
| 120 | THR | CG | 64 | LYS | CE | 3.75 | HCDR2 |  |  |  |  |  |  |  |  |
| 121 | SER | OG | 64 | LYS | NZ | 3.11 | HCDR2 |  |  |  |  |  |  |  |  |

^*^Distances within 4.0 Å between KR01 HA and Fab fragments are shown.


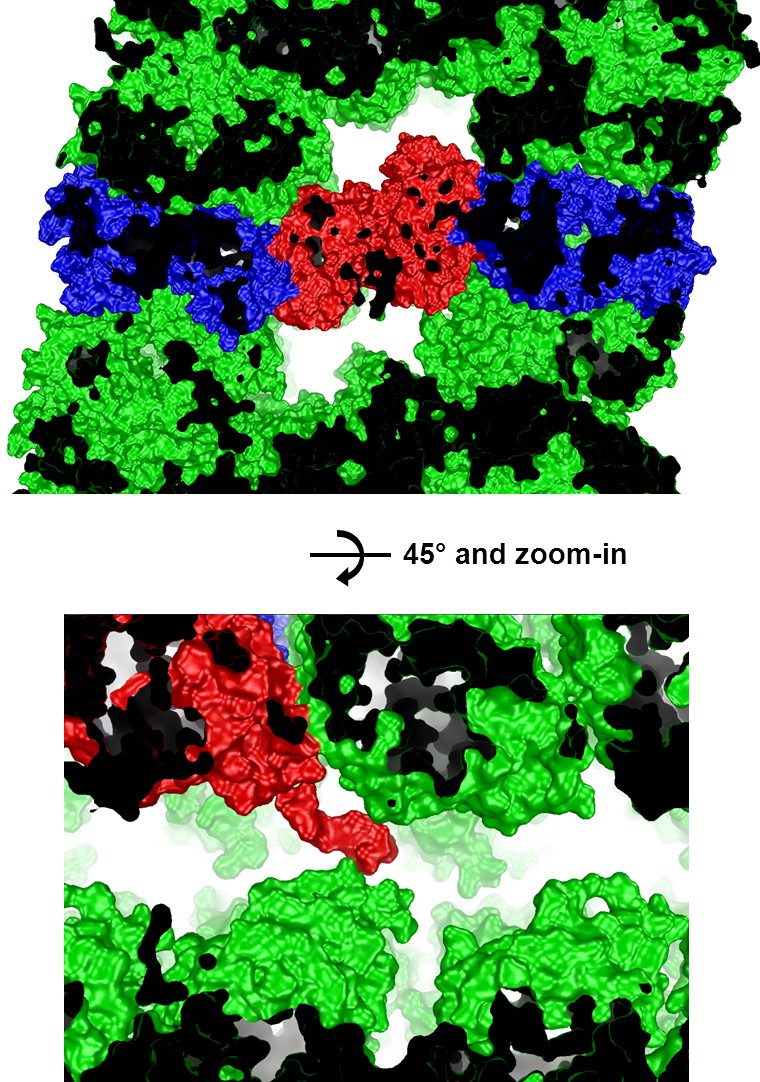


**Figure S1. Crystal packing in the KR01 HA-Fab0587 complex.** The head domain (HA1) of HA is shown in red color and Fab in blue in one complex, and others are in green. The stem regions (HA2) of HA could not be modeled due to weak electron density, but there are empty space for them. A partial model for HA2 is shown in the lower panel.


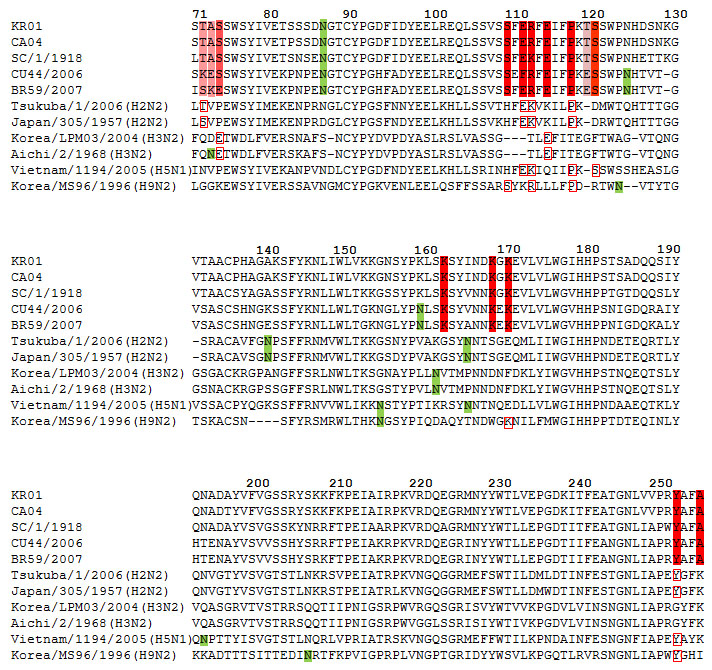


|  |
| --- |
| **Figure S2. Sequence alignments of HAs.** Epitopes in H1 HAs are highlighted in red, and more conserved residues are highlighted in more red. Residues in other HA those are same with that of epitope in H1 HAs are indicated by red boxes. Potential glycosylation sites are highlighted in green. |


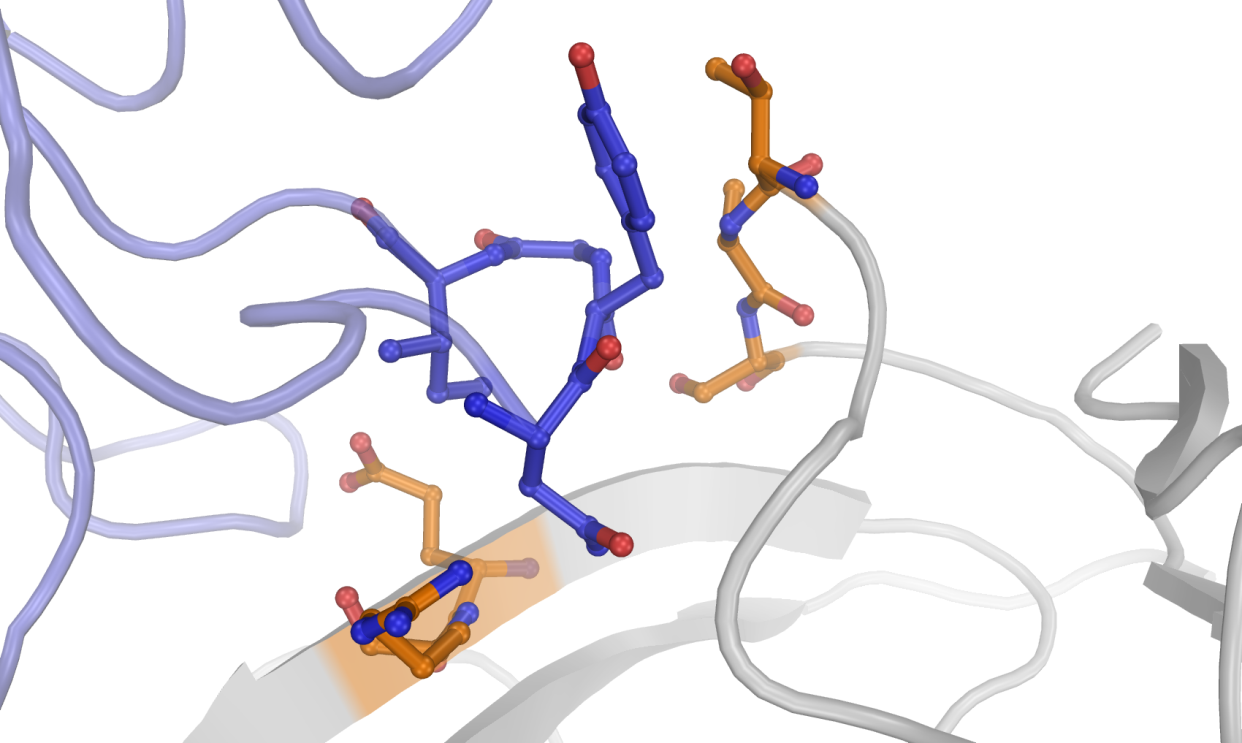


**Arg113**

**Glu112**

**Tyr31**

**Gly32**

**Asn30**

**Ile33**

**Thr72**

**Ala73**

**Ser74**


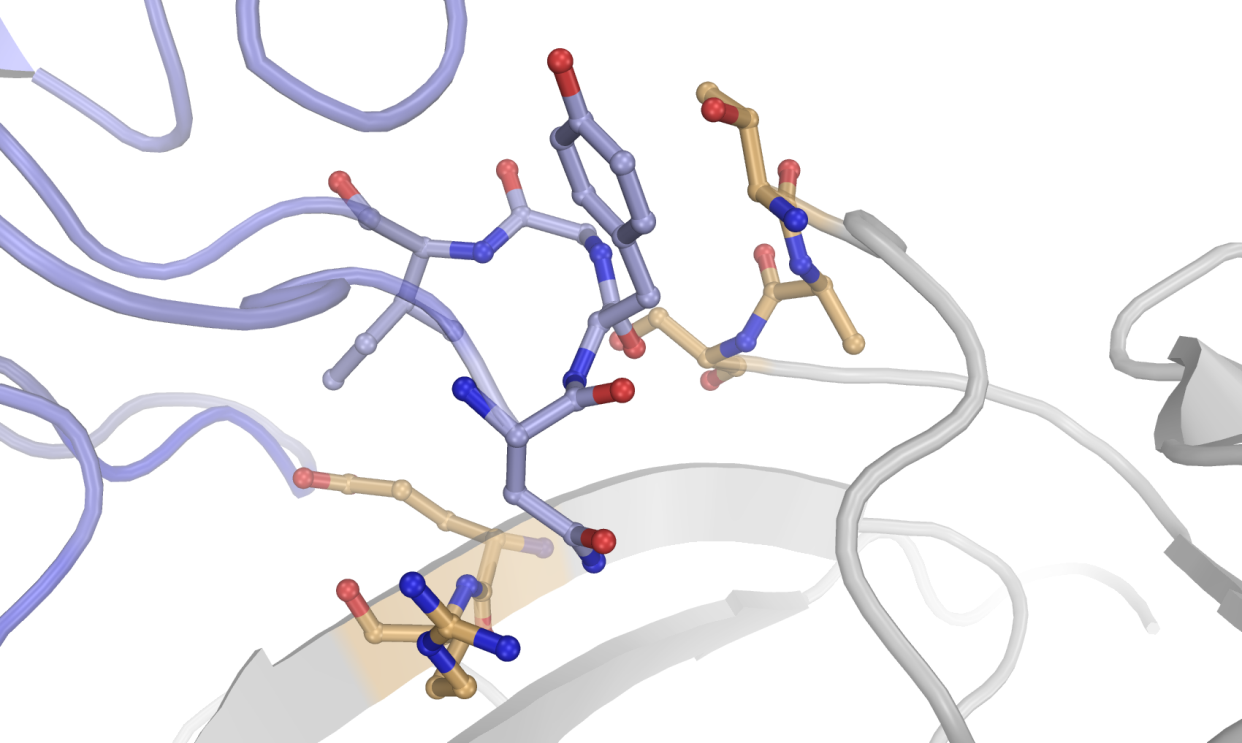


**Arg113**

**Glu112**

**Tyr31**

**Gly32**

**Asn30**

**Ile33**

**Thr72**

**Ala73**

**Ser74**

**Fab0587**

**Fab0757**

**LCDR 1**

**Figure S3.** **LCDR1 at** **Fab binding site.** Residues involved in interactions between HA and Fab0587 are represented as a ball-and-stick model and hydrophilic interactions as dotted line. Residues of HA in complex with Fab0587 are colored in orange and H-chains of Fab0587 and Fab0757 are in dark and light blue, respectively.


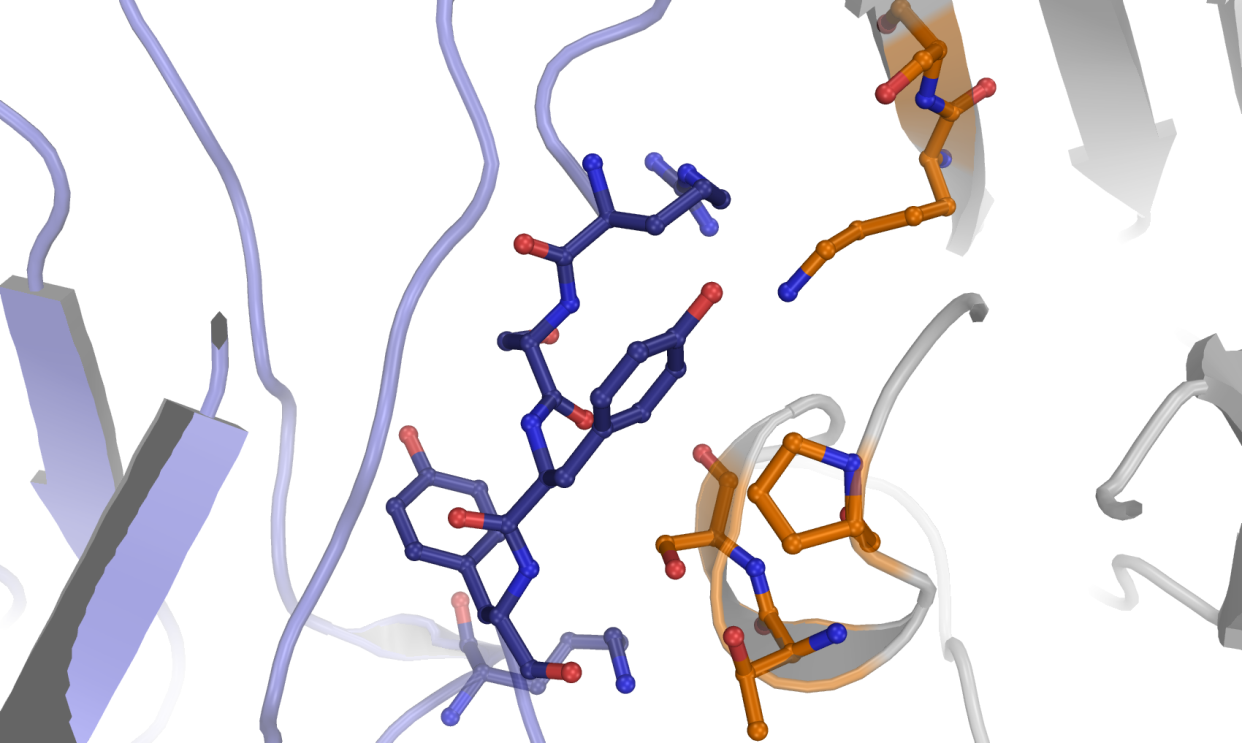


**Tyr59**

**Tyr58**

**Ser57**

**Ser164**

**Ser121**

**Arg56**

**Lys163**

**Pro118**

**Thr120**

**Lys64**


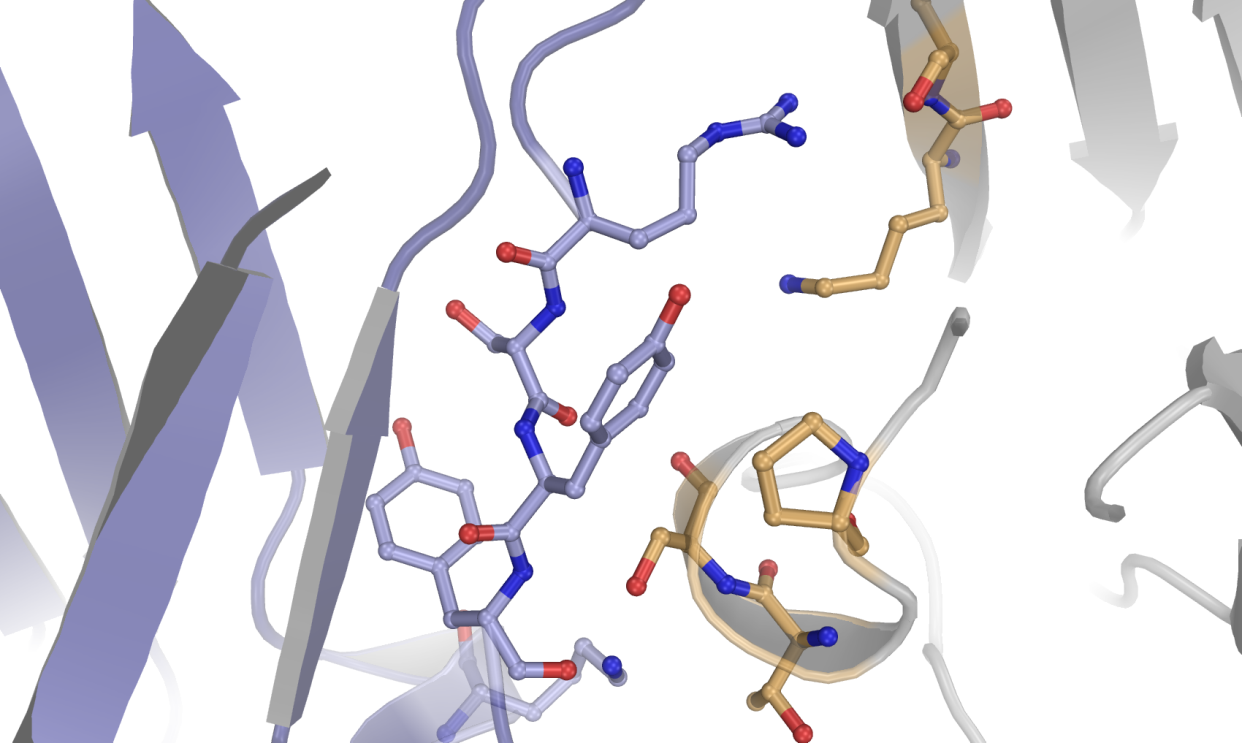


**Tyr59**

**Tyr58**

**Ser57**

**Ser164**

**Ser121**

**Arg56**

**Lys163**

**Pro118**

**Thr120**

**Lys64**

**Fab0587**

**Fab0757**

**HCDR2**

**Figure S4.** **HCDR2 at** **Fab binding site.** Residues involved in interactions between HA and Fab0587 are represented as a ball-and-stick model and hydrophilic interactions as dotted line. Residues of HA in complex with Fab0587 are colored in orange and H-chains of Fab0587 and Fab0757 are in dark and light blue, respectively.

| \| 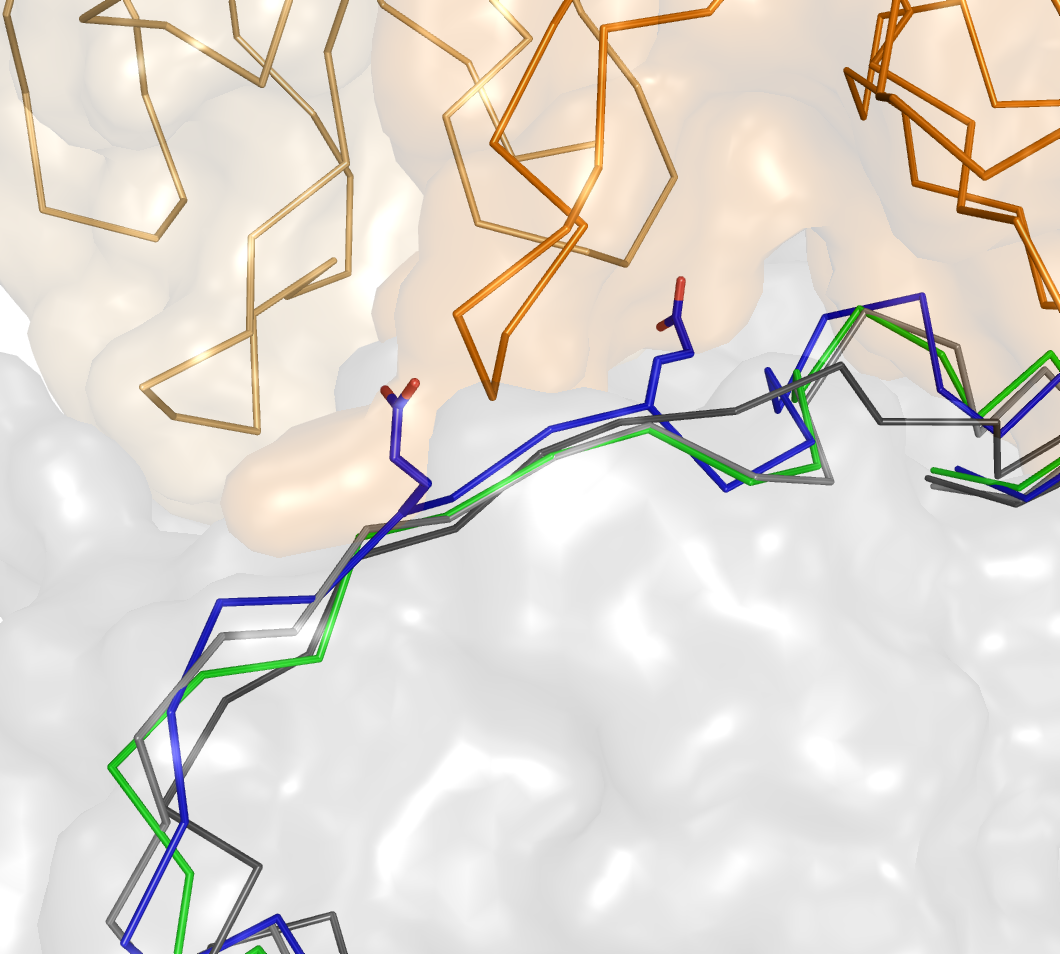 **HCDR3**  **LCDR3**  **LCDR2**  **Glu112**  **Glu115** \| \| --- \| \| **Figure S5. Comparison of novel H1 specific epitope regions among different subtypes.** Superposition of the novel epitope conformations of H1 (blue), H2 (gray), H3 (dark gray), and H5 (green) HA. RMSD was calculated based on secondary structure matching of head domain of H1 with that of other subtypes, to show 1.19, 1.68 and 1.34 Å for H2, H3 and H5, respectively. \| |
| --- | --- | --- |

`
